# Maintenance of Positional Identity of Neural Progenitors in the Embryonic and Postnatal Telencephalon

**DOI:** 10.3389/fnmol.2017.00373

**Published:** 2017-11-13

**Authors:** Ryan N. Delgado, Daniel A. Lim

**Affiliations:** ^1^Department of Neurological Surgery, University of California, San Francisco, San Francisco, CA,, United States; ^2^Eli and Edythe Broad Center of Regeneration Medicine and Stem Cell Research, University of California, San Francisco, San Francisco, CA,, United States; ^3^Biomedical Sciences Program, University of California, San Francisco, San Francisco, CA,, United States; ^4^Medical Scientist Training Program, University of California, San Francisco, San Francisco, CA,, United States; ^5^San Francisco Veterans Affairs Medical Center, San Francisco, CA,, United States

**Keywords:** positional identity, neural development, chromatin regulator, trithorax, polycomb, EZH2, MLL1

## Abstract

Throughout embryonic development and into postnatal life, regionally distinct populations of neural progenitor cells (NPCs) collectively generate the many different types of neurons that underlie the complex structure and function of the adult mammalian brain. At very early stages of telencephalic development, NPCs become organized into regional domains that each produce different subsets of neurons. This positional identity of NPCs relates to the regional expression of specific, fate-determining homeodomain transcription factors. As development progresses, the brain undergoes vast changes in both size and shape, yet important aspects of NPC positional identity persist even into the postnatal brain. How can NPC positional identity, which is established so early in brain development, endure the many dynamic, large-scale and complex changes that occur over a relatively long period of time? In this Perspective article, we review data and concepts derived from studies in *Drosophila* regarding the function of homeobox (Hox) genes, Polycomb group (PcG) and trithorax group (trxG) chromatin regulators. We then discuss how this knowledge may contribute to our understanding of the maintenance of positional identity of NPCs in the mammalian telencephalon. Similar to the axial body plan of *Drosophila* larvae, there is a segmental nature to NPC positional identity, with loss of specific homeodomain transcription factors causing homeotic-like shifts in brain development. Finally, we speculate about the role of mammalian PcG and trxG factors in the long-term maintenance of NPC positional identity and certain neurodevelopmental disorders.

## Positional Identity as a Determinant of Cell Fate

Cell fate determination is a fundamental aspect of metazoan development. At very early stages of mammalian embryogenesis, differences in cell position begin to correspond to distinct developmental fates. For instance, after totipotent blastomeres undergo a process known as compaction (Johnson and McConnell, 2004), cells that are located more superficially give rise to the placenta, while those positioned deeper in the embryo generate the pluripotent progenitors of the inner cell mass (Tarkowski and Wróblewska, 1967; Balakier and Pedersen, 1982; Pedersen et al., 1986; Dyce et al., 1987). As the major axes of the body plan are elaborated, progenitor cells attain more refined positional identities that further correspond to their cell fate decisions (Gilbert, 2010).

**Figure 1:**
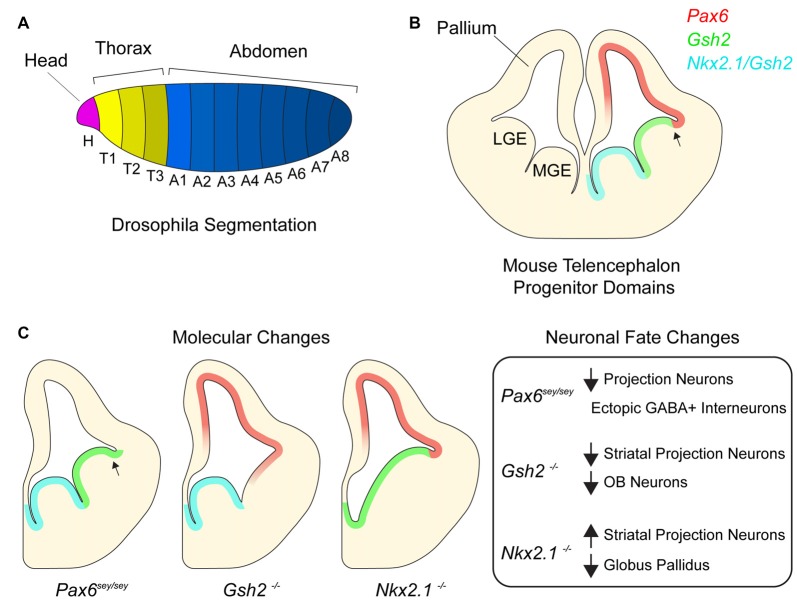
Homeotic transcription factors pattern the developing telencephalon. **(A)** *Drosophila* embryo with different body segments. Different colors of each segment signify different combinatorial expression of homeotic transcription factors. **(B)** Germinal domains and transcription factor patterning in embryonic mouse telencephalon. Medial ganglionic eminence (MGE), lateral ganglionic eminence (LGE). Arrow denotes pallial-subpallial boundary (PSB). **(C)** Schematic depicting molecular changes in various transcription factor mutants and table listing corresponding neuronal fate changes.

In the fruit fly, *Drosophila melanogaster*, the establishment of body plan positional identity can be conceptually divided into two major processes—segmentation and specification. Shortly after gastrulation, the expression of segmentation genes patterns the embryonic ectoderm along the anterior-posterior (AP) axis (Figure 1A; Martinez-Arias and Lawrence, 1985; Akam, 1987). Early segmentation genes (known as gap genes) are responsible for large-scale aspects of the AP axis, while those that are expressed later (pair-rule genes) refine the pattern (Scott and Carroll, 1987). Mutations in segmentation genes result in the loss of segments. For instance, mutations in the gap gene *kruppel* result in a larva missing all thoracic segments and the first five abdominal segments. Pair-rule gene *even-skipped* functions later in segmentation, and its mutation results in the absence of even-numbered segments throughout the length of the AP axis.

Segmentation genes induce the expression of *Drosophila* homeobox (Hox) transcription factors, which in turn specify the identity of each segment (White and Lehmann, 1986; McGinnis and Krumlauf, 1992; Lawrence and Morata, 1994). Mutations in Hox genes do not result in the loss of segment number but rather cause a change in segment identity. For example, the Hox gene *Ubx* is expressed in the third thoracic segment where it is required to generate an appendage called a haltere (Ouweneel and van der Meer, 1973; Lawrence and Morata, 1983). In the absence of *Ubx*, the third thoracic segment fails to generate halters and instead makes an additional wing, an appendage that normally develops from an adjacent segment. This change in segment identity is known as a “homeotic shift” and results from the ectopic expression of *Antp*, a Hox gene responsible for wing development. Such ectopic expression of Hox genes that are normally expressed in neighboring regions is a common transcriptional phenotype of Hox gene mutations. Similar findings have been made in studies of mammalian Hox genes (Pearson et al., 2005) as well as the larger set of homeodomain-containing transcription factors, including those that regulate telencephalic development (Hebert and Fishell, 2008), as we later discuss.

## Polycomb and Trithorax Group Chromatin Regulators and The Maintenance of Positional Identity

The local structure of chromatin—the dynamic polymer of DNA and histone proteins—can influence whether a locus is expressed or silenced. Thus, changes to chromatin structure can engage and maintain particular genetic programs, helping determine cellular identity. The Polycomb group (PcG) and trithorax group (trxG) gene products—which were initially discovered in *Drosophila*—comprise an evolutionarily conserved set of chromatin regulators that appear to serve as a transcriptional “memory” system (Geisler and Paro, 2015; Schuettengruber et al., 2017). By assembling into large multiprotein complexes that modify chromatin structure, PcG and trxG factors help organize the genome regionally into transcriptionally silent or active states, respectively (for a review of PcG and trxG protein molecular mechanisms, please see Steffen and Ringrose, 2014; Geisler and Paro, 2015; Schuettengruber et al., 2017).

In *Drosophila*, shortly after the establishment of Hox gene expression, gap and pair-rule genes are downregulated, and PcG and trxG genes are required to maintain normal patterns of Hox gene expression. For instance, trxG genes are required to maintain the appropriate regional expression of *Ubx* (Kassis et al., 2017). Reminiscent of the homeotic shift observed in *Ubx* mutants, the loss of trxG function results in the development of a second set of wing tissue in place or the normal halters (Breen, 1999). Conversely, PcG genes are required to repress *Ubx* expression in the tissue anterior to the *Ubx* expression domain (Kassis et al., 2017). Of note, Hox gene expression is properly induced in both PcG and trxG mutants but is lost over time (Lewis, 1978; Struhl and Akam, 1985; Yu et al., 1998; Ernst et al., 2004). Thus, in the absence of proper PcG and trxG function, the expression of certain Hox genes is not maintained, resulting in homeotic shifts (Lewis, 1978).

PcG and trxG proteins are also required for the maintenance of Hox gene expression in mice (Schuettengruber et al., 2017). The prototypical trxG gene *trithorax (trx)* and its mammalian homolog *Mixed-lineage leukemia 1 (Mll1)* are both required to positively maintain Hox gene expression (Ingham and Whittle, 1980; Yu et al., 1998). Similar to phenotypes observed in *Drosophila*, Hox gene expression is established normally in *Mll1*-null mice but is not properly maintained, resulting in homeotic transformations of their axial skeleton (Yu et al., 1995, 1998). Homeotic skeletal transformations are also observed in mice null for *Bmi1*, a PcG gene (van der Lugt et al., 1994). Thus, PcG and trxG genes are key components of a “cellular memory system” that maintains the positional identity of progenitor cells in mammalian development.

## Positional Identity in The Embryonic Telencephalon

Similar to regional patterning that occurs in the early embryo, progenitors throughout the developing mammalian central nervous system are organized into distinct domains with different positional identities that are in part defined by the expression of homeodomain transcription factors (Shimamura et al., 1995; Flames et al., 2007; Dasen and Jessell, 2009; Narita and Rijli, 2009; Tümpel et al., 2009). In the embryonic telencephalon, excitatory neurons are born dorsally in the pallium while most inhibitory neurons are born ventrally in the subpallium (Anderson et al., 1997; Puelles et al., 2000; Molyneaux et al., 2007; Kepecs and Fishell, 2014). Pallial progenitors express PAX6 and generate cortical projection neurons which migrate radially and give rise to the six-layered neocortex (Custo Greig et al., 2013). The juxtaposition of PAX6+ progenitors and GSH2+ progenitors of the subpallium forms the pallial-subpallial boundary (PSB; Toresson et al., 2000; Corbin et al., 2003). The subpallium is further subdivided into several subdomains including the lateral and medial ganglionic eminences (LGE and MGE), which generate different subtypes of inhibitory neurons. The LGE is located immediately ventral to the pallium and is dorsal to the MGE. LGE progenitors give rise to a large number of olfactory bulb (OB) interneurons and striatal projection neurons while the MGE generates cortical interneurons and the globus pallidus as well as a small number of OB interneurons (Butt et al., 2008; Xu et al., 2008; Flandin et al., 2010). *Nkx2.1* is expressed throughout the MGE and not detected in the LGE. Together, the pallium, LGE and MGE form three molecularly distinct domains along the dorsoventral axis of the embryonic telencephalon (Figure 1B). In addition to their expression defining the location and regional boundaries of these domains, *Pax6*, *Gsh2* and *Nkx2.1* are also required to specify the developmental potential of neural progenitor cells (NPCs) in their respective domains, as we discuss below.

## Homeotic-Like Shifts in The Developing Brain

*Small eye* (*Sey*) is a naturally occurring *Pax6* allele that is a loss-of-function nonsense mutation (Hill et al., 1991). While the developing neocortex of *Pax6*^Sey/Sey^ mice maintains the expression of certain pallial genes such as *Tbr1* and *Math2*, some NPCs gradually become mis-specified, adopting a subpallial-like identity (Figure 1C; Manuel et al., 2015), with *Gsh2* and subpallial genes *Ascl1* and *Dlx2* becoming expressed dorsally across the PSB (Toresson et al., 2000). Furthermore, by E15.5, pallial NPCs in* Pax6*^Sey/Sey^ mice begin generating GABAergic interneurons with an LGE-like identity (Kroll and O’Leary, 2005). These data suggest that as the neocortex develops, sustained *Pax6* expression is required to repress ventral telencephalic gene expression and associated neuronal fates.

In the subpallium, *Gsh2* is critical to the specification of LGE positional identity. In the LGE of E12.5 *Gsh2*^−/−^ mice, the expression of *Ascl1* and* Dlx2* is nearly undetectable (Szucsik et al., 1997; Corbin et al., 2000; Toresson et al., 2000; Yun et al., 2001). Furthermore, expression of *Pax6* as well other pallial genes (e.g., *Tbr2* and *Ngn2*) extends ventrally past the PSB into the dorsal LGE. This early absence of LGE identity and ectopic expression of pallial genes correlates with the development of a smaller striatum, and embryonic OB neurogenesis is also impaired. Thus, without *Gsh2*, the dorsal LGE initially takes on a pallial-like NPC identity, and the genesis of LGE-lineage neuronal subpopulations is defective (Figure 1C; Corbin et al., 2000; Yun et al., 2001).

*Nkx2.1* plays key roles in the positional identity of the MGE. In the absence of *Nkx2.1* expression, the MGE adopts an LGE-like identity and fails to generate MGE-specific neuron populations (Figure 1C; Sussel et al., 1999; Butt et al., 2008; Nóbrega-Pereira et al., 2008). For instance, conditional deletion of *Nkx2.1* at E10.5 from the subpallium decreases the production of MGE-derived cortical interneurons (Butt et al., 2008), and in *Nkx2.1*^−/−^ mice, development of the globus pallidus is severely impaired (Sussel et al., 1999). Without *Nkx2.1*, the mutant MGE appears to become partially dorsalized, having ectopic expression of the normally LGE-specific transcription factors *Isl1*, *SCIP* and *GOLF*. Consistent with this LGE-like transcriptional character, the *Nkx2.1*-null MGE generates striatal neurons. Furthermore, conditional deletion of *Nkx2.1* at E16 results in the loss of chandelier cells (Taniguchi et al., 2013), a later-born population of cortical interneurons (Inan et al., 2012). Taken together, these data indicate that *Nkx2.1* is required for the maintenance of MGE identity and proper developmental potential of this population of ventral NPCs in both the early and late embryonic brain.

## NPCs in The Postnatal Brain: Maintenance of Embryonic Positional Identity

The postnatal mammalian brain harbors NPCs in the ventricular-subventricular zone (V-SVZ), a layer of cells found along the walls of the cerebral ventricles (Lim and Alvarez-Buylla, 2016). In the adult mouse brain, V-SVZ NPCs—known as B1 cells—give rise to neuroblasts that migrate to the OB where they differentiate into several types of interneurons. The two main categories of OB interneurons are granule cells (GCs) and periglomerular cells (PGCs), both of which can be further divided into additional subtypes (Price and Powell, 1970; Kosaka et al., 1997). GC interneurons can be categorized as superficial or deep depending on their location within the GC layer. PGCs can be divided into three mutually exclusive groups by their expression of Calbinden (CalB), Calretinin (CalR), or tyrosine hydroxylase (TH).

**Figure 2:**
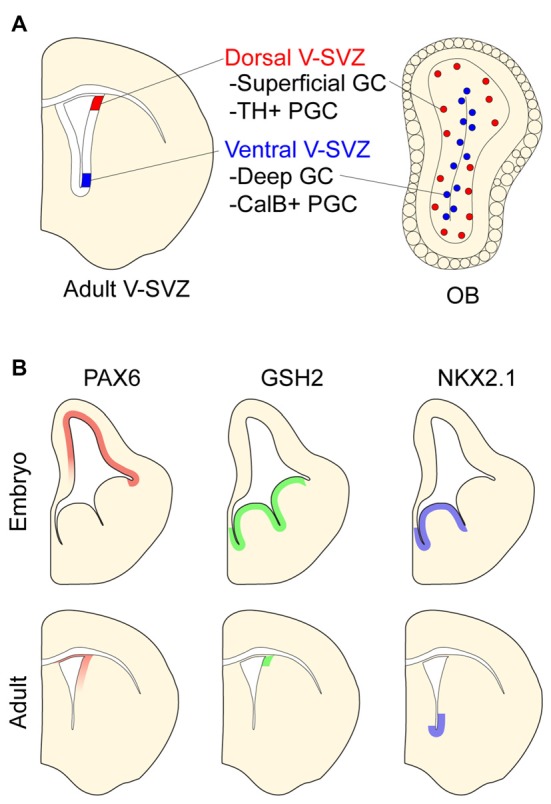
Positional identity in the postnatal ventricular-subventricular zone (V-SVZ). **(A)** Schematic of postnatal V-SVZ and corresponding olfactory bulb (OB) section depicting positional identity of V-SVZ neural stem cells. Deep (blue) and superficial (red) granule cells (GCs) depicted in OB. **(B)** Regional transcription factor patterning in embryonic and postnatal V-SVZ germinal zones. Boundaries of protein expression depicted in both embryonic and postnatal germinal zones.

Similar to embryonic NPCs, B1 cells have distinct positional identities that give rise to different subtypes of PGCs and GCs. For instance, while B1 cells in the dorsal V-SVZ produce superficial GCs and TH+ PGCs, ventral B1 cells generate deep GCs and CalB+ PGCs (Figure 2A; Merkle et al., 2007; Alvarez-Buylla et al., 2008; Rushing and Ihrie, 2016). Interestingly, such regional differences in the developmental potential of V-SVZ NPCs are retained even when serially passaged *in vitro* and transplanted into different locations of this postnatal germinal zone. For example, ventrally derived NPCs transplanted to the dorsal V-SVZ still produce deep granule neurons but not TH-positive PGCs (Merkle et al., 2007). Thus, regional differences in V-SVZ NPCs appear to be in large part cell-intrinsic and stable through serial cell divisions.

Postnatal B1 cells arise from embryonic NPCs (Merkle et al., 2004; Young et al., 2007; Delgado and Lim, 2015). Clonal analysis with a “barcoded” retroviral library demonstrates that B1 cells share a common embryonic NPC with those that generate neurons for the cortex, striatum and septum (Fuentealba et al., 2015). When NPCs are transduced with retroviral vectors at E12.5 and brains analyzed after ~5 weeks, approximately 35% of the clones contain postnatally-born OB neurons as well as embryonically-born forebrain cells. Further analysis of such clones suggests that B1 cells retain positional information of the shared embryonic NPC. For example, clones containing cortical projection neurons (indicating their birth from pallial NPCs) include superficial OB GCs (which arise from B1 cells close to the pallium). Thus, the positional identity of V-SVZ NPCs appears to be established during embryogenesis and persists throughout development and into postnatal life.

Like the embryonic germinal zones, the postnatal V-SVZ exhibits regional patterns of transcription factor expression that correspond to the developmental potential of local NPCs (Figure 2B and reviewed in Alvarez-Buylla et al., 2008). For instance, the dorsal V-SVZ expresses *Emx1*, and consistent with the developmental potential of B1 cells in this region (Merkle et al., 2007), *Emx1*-lineage cells predominantly generate TH+ and CalR+ PGCs in adulthood (Fuentealba et al., 2015). In the most ventral aspect of the V-SVZ, B1 cells express *Nkx2.1*. Administering tamoxifen to adult Cre-reporter mice carrying the *Nkx2.1*-CreER “knock-in” allele labels ventral B1 cells that produce deep OB GCs, which is coherent with results from stereotactic methods of labeling ventral B1 cells (Merkle et al., 2007). Several other regional V-SVZ subdomains defined by the expression of specific transcription factors have been similarly defined and found to generate region-appropriate OB subtypes (Merkle et al., 2014).

The pattern of regional transcription factor expression in the V-SVZ is similar to that observed in embryonic development (Alvarez-Buylla et al., 2008). Interestingly, for some of these genes, it appears that such regional expression is maintained in NPCs throughout embryonic development and into adulthood. For example, pallial NPCs labeled with *Emx1*-CreER at E10.5 give rise to cells that populate the dorsal V-SVZ (Young et al., 2007) where *Emx1* continues to be expressed postnatally. Similarly, MGE NPCs labeled at E12.5 with *Nkx2.1*-CreER give rise to ventral V-SVZ cells including local B1 cells (Delgado and Lim, 2015). Of note, virtually all V-SVZ cells expressing the Cre-reporter are also immunopositive for NKX2.1 protein in adult mice. Even when serially passaged in culture, ventral V-SVZ NPCs retain the expression of *Nkx2.1* (Delgado et al., 2016). These data suggest that key transcriptional differences that define the positional identity of B1 cells relate to the “retention” of region-specific gene expression that was established very early in brain development.

## Potential Mechanisms Underlying The Maintenance of NPC Positional Identity

The persistence of regionally discrete gene expression along the developmental continuum of NPCs from the early embryo to the adult is remarkable not only in terms of duration, but also because of the tremendous increase in size and anatomic complexity of the brain over this period of time. How is NPC positional identity maintained in the face of these challenges? Below, we touch upon the potential roles of morphogens, transcriptional feedback mechanisms, non-coding RNAs (ncRNAs) and the mammalian PcG/trxG chromatin regulators. To simplify the context of this brief discussion, we focus on the population of *Nkx2.1+* NPCs.

The establishment of regional transcription factor expression in the embryonic telencephalon requires the actions of morphogens such as *Sonic hedgehog* (*Shh*; Wilson and Rubenstein, 2000; Monuki and Walsh, 2001; Hebert and Fishell, 2008). Shh is required for the early induction of *Nkx2.1* in the ventral neural tube, and at E12.5, Shh appears to maintain *Nkx2.1* expression in the MGE (Xu et al., 2005; Gulacsi and Anderson, 2006). Given that genes downstream of Shh signaling are expressed in the postnatal V-SVZ (Ihrie et al., 2011), it is possible that Shh is also required to maintain *Nkx2.1* in ventral B1 cells. Deletion of *Shh* in adulthood reduces the production of ventrally derived OB interneurons (Ihrie et al., 2011), suggestive of a loss of ventral NPC identity. However, Shh is also a mitogen for NPCs (Palma et al., 2005), and it remains to be determined if changes in the proliferation of V-SVZ cells contribute to these findings. Can morphogens alone be expected to maintain regionally discrete gene expression as the brain grows in size and anatomic complexity? Though certainly conjecture, we suggest that biological mechanisms other than Shh signaling are required to maintain *Nkx2.1* expression at some point along the developmental continuum of early embryonic to postnatal NPCs.

Transcriptional autoregulation is an important mechanism underlying the maintenance of homeotic gene expression during embryogenesis (Lou et al., 1995; Packer et al., 1998). The *Nkx2.1* promoter region contains conserved NKX2.1-binding sites to which NKX2.1 can bind and positively regulate transcription (Oguchi and Kimura, 1998; Das et al., 2011). Thus, it is possible that NKX2.1 uses a positive feedback loop for transcriptional maintenance, which could also contribute to the “discreteness” of *Nkx2.1* expression. While transcription of the *Nkx2.1* locus does not require functional NKX2.1 protein (mutant transcripts are detected in *Nkx2.1*^−/−^ NPCs; Sussel et al., 1999; Toresson et al., 2000), whether expression of the mutant alleles diminishes over time has not been reported. In any case, transcriptional autoregulation (as well as additional regulatory mechanisms such as the activation or repression of other genes) likely requires the function of chromatin regulatory factors.

The mammalian genome transcribes a large number and diversity of ncRNAs, and specific ncRNAs can regulate the expression of Hox transcription factors. Long noncoding RNAs (lncRNAs) are transcripts longer than 200 nucleotides that do not code for protein, and it is now clear that certain lncRNAs have important cellular function and interact with PcG/trxG factors (Davidovich and Cech, 2015; Engreitz et al., 2016). In the developing brain, many lncRNAs are highly cell-type specific (Liu et al., 2016), and some lncRNAs play key roles in neurodevelopment (Andersen and Lim, 2017). In the mouse genome, the lncRNA NANCI is located adjacent to *Nkx2.1* and is co-expressed with *Nkx2.1* in the lungs where it positively regulate *Nkx2.1* transcription (Herriges et al., 2014, 2017). In the forebrain including the MGE, NANCI is also co-expressed with *Nkx2.1* (Herriges et al., 2014), but whether NANCI helps maintain *Nkx2.1* expression in a stable and heritable manner throughout development has not been reported.

microRNAs (miRNAs) are an important class of ncRNAs that downregulate gene expression post-transcriptionally via base-paring with complementary sequences within the target mRNA transcript (Bartel, 2009). Some miRNAs target the mRNAs of Hox transcription factors and may thus play roles in NPC positional identity. For instance, miR-7a is expressed in a ventral-to-dorsal gradient in the mouse V-SVZ, and this miR-7a gradient contributes to the regional expression of PAX6 protein (de Chevigny et al., 2012). While miR-365 has been shown to negatively regulate *NKX2.1* in lung cancer cell lines (Kang et al., 2013) its potential role in regulating *Nkx2.1* expression in the forebrain has not been reported. Importantly, given that miRNAs can regulate the expression of PcG genes such as EZH2 (Szulwach et al., 2010; Neo et al., 2014), and that some miRNAs are embedded within the *Hox* gene clusters (Mansfield et al., 2004; Tehler et al., 2011), it will be important to consider the role of miRNAs in NPC positional identity.

Most studies investigating the role of mammalian PcG and trxG chromatin regulators in neural development have focused on the general processes of NPC self-renewal, neuronal differentiation and gliogenesis (reviewed in Hirabayashi and Gotoh, 2010; Lim and Alvarez-Buylla, 2014). However, given that PcG/trxG proteins play key roles in the maintenance of Hox gene expression and positional identity during development of the axial body plan, it seems reasonable to consider the possibility that these factors also maintain the positional identity of NPCs during brain development. For instance, similar to the requirement for *Mll1* in the maintenance of Hox gene expression of the early mouse embryo (Yu et al., 1995), does the continuous expression of *Nkx2.1* and related ventral identity of NPCs also depend on the action of mammalian trxG proteins? More generally, would deficiencies of trxG or PcG activity lead to homeotic-like shifts in telencephalic NPCs, changing the proportions of neuronal subtypes that are produced from each region?

## The Importance of Understanding How NPCs Maintain Positional Identity

Current concepts regarding NPC positional identity in the mammal primarily involve morphogens (or other inductive signals) and transcription factors. Based on data and concepts described in sections above, we suggest that certain chromatin regulators are integral to the maintenance of NPC positional identity. That is, while morphogen gradients indeed establish regional patterns of NPCs in the early telencephalon, and transcription factor regulatory networks are likely critical to such positional identity, certain chromatin regulators may be required for NPCs to “remember” key region-specific aspects of their transcriptome as they continue to proliferate throughout development. The notion that chromatin regulators are critical to NPC positional identity (and thus their developmental potential) may have important implications for our understanding of human disease as well as the culture of NPCs for human therapeutics and drug development.

The maintenance of NPC positional identity is likely crucial to proper brain development, as loss of such developmental information might be expected to cause homeotic-like shifts that result in abnormal neuroanatomy. Recent studies have implicated mutations in many chromatin regulators as causes of human neurodevelopmental and psychiatric disorders (Ronan et al., 2013; De Rubeis et al., 2014). For instance, *EZH2* and *MLL1* are mutated in Weaver syndrome (Tatton-Brown et al., 2011) and Weidemann-Steiner syndrome (Jones et al., 2012; Strom et al., 2014), respectively, both of which are associated with intellectual disability. Understanding the potential role that these PcG and trxG factors play in NPC positional identity may provide important insights into the pathology of certain neurodevelopmental disorders.

The ability to produce specific neural cell types from cultured NPCs is broadly useful for research into human neurological disease and therapeutic development. The types of neurons generated from NPCs relates to their positional identity, which is generally induced by the application of morphogens. Discovering the mechanisms by which proliferating NPCs retain specific transcriptional programs for long periods of time may thus enhance our ability to durably propagate specific types of NPCs *in vitro* for such translational purposes. Furthermore, such research may inform methods by which we can “erase” and re-establish NPC identity.

## Concluding Remarks

It is now clear that the positional identity of neural progenitors is an important contributor to neuronal diversity. As discussed above, the processes regulating NPC positional identity are reminiscent of those that regulate segment identity in the developing *Drosophila* larvae. As for progenitor cells of *Drosophila* larval segments, the positional identity of telencephalic NPCs requires the sustained expression of homeodomain transcription factors. Loss of such region-specific fate regulators causes embryonic NPCs to become misspecified and adopt the identity of the adjacent region, which results in abnormal anatomy. The mechanisms required for the maintenance of NPC positional identity are poorly understood at this time, but our understanding of chromatin regulators such as PcG and trxG factors can be incorporated into current concepts related to morphogen signaling and transcription factor networks. Given the particularly long duration of NPC proliferation in human brain development, the maintenance of NPC positional identity will likely have important implications to our understanding of certain neurodevelopmental disorders.

## Author Contributions

RND and DAL wrote the perspective article. Some parts of this manuscript have been adopted from RND’s Doctoral Thesis.

## Conflict of Interest Statement

The authors declare that the research was conducted in the absence of any commercial or financial relationships that could be construed as a potential conflict of interest.
